# Integrating pedagogical content knowledge and pedagogical/psychological knowledge in mathematics

**DOI:** 10.3389/fpsyg.2014.00924

**Published:** 2014-08-20

**Authors:** Nora Harr, Andreas Eichler, Alexander Renkl

**Affiliations:** ^1^Department of Psychology, University of FreiburgFreiburg, Germany; ^2^Department of Mathematics, University of EducationFreiburg, Germany

**Keywords:** teacher education, higher education, instructional design, pedagogical content knowledge, general pedagogical/psychological knowledge

## Abstract

In teacher education at universities, general pedagogical and psychological principles are often treated separately from subject matter knowledge and therefore run the risk of not being applied in the teaching subject. In an experimental study (*N* = 60 mathematics student teachers) we investigated the effects of providing aspects of general pedagogical/psychological knowledge (PPK) and pedagogical content knowledge (PCK) in an integrated or separated way. In both conditions (“integrated” vs. “separated”), participants individually worked on computer-based learning environments addressing the same topic: use and handling of multiple external representations, a central issue in mathematics. We experimentally varied whether PPK aspects and PCK aspects were treated integrated or apart from one another. As expected, the integrated condition led to greater application of pedagogical/psychological aspects and an increase in applying both knowledge types simultaneously compared to the separated condition. Overall, our findings indicate beneficial effects of an integrated design in teacher education.

## INTRODUCTION

For quite some time, there has been criticism concerning the imbalance in teacher education between university education and later job demands (Finn, 2001; Grossman, 2008). One of the main objections voiced is the current organization of teacher education that separates *subject matter knowledge* [i.e., *content knowledge* and *pedagogical content knowledge* (*PCK*); see Kleickmann et al., 2013] from pedagogy. In pedagogy, student teachers typically acquire knowledge of domain general pedagogical and psychological aspects, providing them with important knowledge for powerful teaching (Voss et al., 2011). However, these methods courses are rarely connected to content of teaching or subject didactics (e.g., mathematics education), leaving the challenge of integration to the individual teacher.

This fragmented practice is based on the (implicit) assumption that integration is simple and builds up automatically. However, when teachers are obliged to integrate different pieces of knowledge by themselves, this demand creates considerable difficulty and integration often hardly occurs (Ball, 2000). In other words, knowledge taught in different courses, at different times, or by different departments hardly becomes integrated. Hence, *pedagogical/psychological knowledge* (i.e., *PPK*; Voss et al., 2011), content knowledge, and PCK (Shulman, 1986, 1987) are at risk of being encoded in different cognitive compartments without substantial cross-referencing (Renkl et al., 1996). This education-made chasm can lead to inert knowledge (Whitehead, 1929) - the non-use of general PPK when teaching certain content areas. General PPK, however, can be regarded as an important prerequisite to creating and optimizing teaching situations (Grossman and McDonald, 2008; Voss et al., 2011). For example in mathematics, the domain-general pedagogical/psychological subdimensions “teaching methods” and “knowledge about learning processes and individual characteristics” become especially important as soon as teachers incorporate multiple representations, a common practice in mathematics lessons.

The present study experimentally analyzes the effects of providing subdimensions of general PPK and PCK in an integrated or separated way by comparing two computer-based teaching conditions. The separated condition treats aspects of general PPK and PCK apart from one another. The integrated condition contains both knowledge types in an interrelated way.

## TEACHING KNOWLEDGE NEEDED FOR MATHEMATICS

Competent teaching is based on complex interaction between multiple types of knowledge stemming from various sources (Bromme, 1997; Segall, 2004; Ball et al., 2008). In order to account for the psychological and pedagogical aspects of pedagogical knowledge, Voss et al. (2011) broadened Shulman’s original definition. They extended general pedagogical knowledge to include pedagogical and psychological aspects and defined specific subdimensions (i.e., classroom management, teaching methods, classroom assessment, knowledge about learning processes, and individual characteristics). They introduced the term general PPK which comprises knowledge of teaching-learning situations that is applicable across different teaching subjects.

In addition to content and general PPK, PCK is crucial for good teaching (Shulman, 1986; Grossman and Richert, 1988; Borko and Putnam, 1996; Ball, 2000; Ball et al., 2008). Considering the widespread belief that professional teacher knowledge is based on different types of knowledge (Bromme, 1997; Ball et al., 2008), it is surprising that teacher education is usually divided into subject matter knowledge (i.e., content knowledge and PCK) and pedagogy (Ball, 2000). This separation may lead to knowledge compartmentalization of main parts of PPK and PCK, that is, both knowledge types risk being stored with little reference to each other in largely unconnected memory parts. Such knowledge compartmentalization need not always pose a problem. When teachers act in well-known content areas, they can rely on their well-tailored PCK (Shulman, 1986; Borko and Putnam, 1996). However, when teachers act in unfamiliar or even “new” contents for which they have no or very little PCK, they seem to apply their general PPK (Hashweh, 1987). If this PPK has been acquired in separate university courses (e.g., on educational psychology) that made no or few references to the teaching domain (e.g., mathematics), the acquired pedagogical/psychological principles can hardly be transformed into effective action within a domain such as mathematics (see Renkl et al., 1996; Gruber et al., 2000). To foster applicable PPK, we suggest that this type of knowledge is taught with crosslinks to content-related topics.

We assume that an integrated teaching approach has two main advantages. First, principles get “filled” with domain-specific content, thus an integrated encoding of pedagogical/psychological principles with domain-specific examples is supported (e.g., which and how multiple representations are typically used in a particular content domain). Such integrated encoding should render PPK applicable, as suggested for example by Renkl’s (2014) theory of example-based learning or Bandura sub-theory on abstract modeling (Bandura, 1986). A second advantage of integration - the simultaneous application of knowledge types - refers to situations in classroom teaching where switching back and forth between different knowledge types can be useful. Following the assumption of *spreading activation* (e.g., Anderson, 1983), integrating knowledge types should promote a simultaneous retrieval of knowledge due to the association of them. In this view, memory is described as a web of associated knowledge pieces (i.e., nodes). Once a node (e.g., representing PCK) is activated, activation transfers along to related nodes (e.g., representing PPK).

It is conceivable that the integration of different knowledge types does not only have advantages (e.g., Ainsworth et al., 2002; Ainsworth, 2006; Ayres, 2013; Schwonke et al., 2013). The simultaneous learning of various knowledge types can prove overwhelming to pre-service teachers (Brush and Saye, 2009). Learners (e.g., student teachers) are required to repeatedly shift between topics (e.g., PCK and PPK) and to integrate them. When confronted with complex material from two origins and with shifting and integration demands, working memory may experience a heavy load or even overload (Sweller et al., 2011). Thus, learning can then be hindered. The danger of overloading learners is especially prevalent when they have low working memory capacities (e.g., Engle, 2002; Baddeley, 2012). In this context, it is also important to note that cognitive load is also determined by learners’ prior knowledge (Sweller et al., 1998). When novice learners with little prior knowledge encounter new information units, they have to process them as separate entities in their working memories (Kalyuga, 2008). In contrast, more advanced learners have acquired schemata allowing them to cluster related single information units within larger chunks which can then be treated as single entities (Ericsson and Kintsch, 1995; Kalyuga, 2008). Hence, in particular low prior-knowledge learners might be overwhelmed by the demand to integrate different types of knowledge.

## THE PRESENT STUDY

The present study experimentally tested the effects of providing aspects of general PPK and PCK (here, about mathematics education) in an integrated or separated way. As instruction can be regarded an important activity for teachers, we focused on two instruction-relevant subdimensions of PPK as well as two corresponding subdimensions of PCK (see **Table 1**). We illustrated these aspects of general PPK and PCK (i.e., mathematics education-specific knowledge) on the topic of multiple representations in mathematics instruction because they are highly relevant in this domain.

**Table 1:** Overview of the subdimensions focused in the learning environments.

Subdimension	PPK	PCK
(1)	“Teaching methods” (Voss et al., 2011, p. 953; see also Borko and Putnam, 1996: “instructional strategies for conducting lessons and creating learning environments”, p. 675)	“Knowledge of strategies and representations for teaching particular topics” (Borko and Putnam, 1996, p. 677; see also Ball et al., 2008: “knowledge of content and teaching”, p. 401)
(2)	“Knowledge about learning processes and individual characteristics” (Voss et al., 2011, p. 953; see also Borko and Putnam, 1996: “knowledge and beliefs about learners, how they learn and how that learning can be fostered by teaching”, p. 675)	“Knowledge of students’ understandings [… and of] how students learn in a particular content domain” (Borko and Putnam, 1996, p. 676; see also Ball et al., 2008: “knowledge of content and students”, p. 401)

In mathematics, teachers frequently use texts, formulas, tables, and graphs. Often the same or at least overlapping information is displayed in varying representations (e.g., fractions in a pie chart or as decimal number). The flexible use of such representations plays an important role in learning mathematics (Heinze et al., 2009). Both, American and German standards of education stress the importance of multiple representations (e.g., National Council of Teachers of Mathematics, 2000; Bildungsstandards, 2004; National Board for Professional Teaching Standards, 2013). Students should be able to flexibly use representations, communicate mathematical ideas through them, select, apply, and translate among them, and to interconnect them.

Although multiple external representations can support learning (Ainsworth, 1999; Eitel et al., 2013), psychologically oriented research has shown that processing them appropriately is very demanding (Schnotz and Bannert, 2003; Ainsworth, 2006; Seufert and Brünken, 2006; Berthold and Renkl, 2009). Especially the transition and integration processes between representations pose a crucial obstacle to learners (Duval, 2006). In fact, many learners tend to use multiple external representations suboptimally in that they only use a few familiar ones or fail to integrate them (Schwonke et al., 2009).

The present study analyzed the effects of providing knowledge about multiple representations in an integrated or separated way. More specifically, we addressed the following four research questions. Firstly, we tested whether the integration of knowledge types would enhance the applicability of PPK concepts. At the meantime, we did not expect integration to yield beneficial effects in applying aspects of PCK, because we assumed that due to them already being content-related, no major inertia problem exists which could be overcome by combining different knowledge types. We thus expected participants to be able to apply general PPK better if they learn general PPK and PCK in an integrated as compared to a separated way (Hypothesis 1). Secondly, we assumed that an integrated presentation facilitates switching back and forth between content-specific and content-independent considerations when thinking about teaching problems. Accordingly, we predicted that the integrated condition is superior in applying both perspectives simultaneously (i.e., aspects of PPK and PCK) to specific teaching problems (Hypothesis 2). Thirdly, as participants with low prior knowledge or low working memory capacity might be overwhelmed by an integrated presentation, we assumed aptitude–treatment interaction effects: The positive effects of integration are moderated by prior knowledge (Hypothesis 3) and working memory capacity (Hypothesis 4).

## MATERIALS AND METHODS

This empirical study was performed in accordance with the German Psychological Society (DGPs) ethical guidelines (2004, CIII) and the APA ethical standards. The German Psychological Society’s ethical commission states that approval from an institutional research board only need be obtained if funding is subject to the ethical approval by an Institutional Review Board. This research was reviewed and approved by the Ministry of Science, Research, and Arts of Baden-Württemberg, Germany [grant number 7532.3/130], which did not require additional Institutional Review Board approval. The Ministry of Science, Research, and Arts of Baden-Württemberg, Germany approved the research procedures of this study. The participants volunteered to participate and received a compensation of 15 Euros for participating. All participants were aware of taking part in research. Before starting, a standardized explanation about ethical guidelines was read out loud and participants provided verbal informed consent. Participants who declined to provide the verbal informed consent were offered the possibility to withdraw from the experiment and still receive the financial compensation. All participants provided written informed consent allowing us to use their collected data anonymously for publications. All data was anonymously collected and analyzed.

### PARTICIPANTS AND DESIGN

Mathematics student teachers (*N* = 60, 33 females) from a German university volunteered to participate in this study. The incentive was 15 Euros, disbursed immediately after participation. Their average age was 21.15 years (SD = 1.57). Most of them were in their second or fourth semester of teacher training (76.3%). They were recruited in various mathematics lectures and via posters at the Institute of Mathematics over a period of one month. After registration, the participants were randomly assigned to one of the two learning conditions and participated in a group session where they worked individually on computers. In the first condition, they received aspects of PPK and PCK on the topic of multiple external representations successively in a separated way (“separated condition”, *n* = 29). In the second condition, the knowledge types (i.e., PPK and PCK aspects on multiple external representations) were provided in a combined, integrated way (“integrated condition”, *n* = 31). Dependent variables comprised two scores, one for the application of PPK and one for the PCK aspects, as well as measures of the combined use of knowledge types. One participant in the separated condition had to be excluded from further analyses due to inappropriate behavior during data collection and unevaluable/invalid answers.

### MATERIALS

#### Working memory task

We assessed the working memory span of each participant individually. This task was designed in accordance with the reading span task described by Unsworth et al. (2005). The participants read and subsequently classified sentences to be either sensical or nonsensical. While doing so, they were asked to remember a set of unrelated letters presented at the end of each sentence (e.g., “The infant suffered from an ear infection and therefore had to stay in the lettuce for three weeks. X”). For working memory scores to be valid, a threshold of 85% accuracy in sentence-classification was required to ensure that participants were not trading off between reading the sentence and remembering the letters. The amount of sensical and nonsensical sentences was balanced and the sentences 10–15 words long. Nonsensical sentences were created by replacing a single word in an otherwise sensical sentence. The sentences were presented in sets, varying from two to five sequenced sentences. Following each set, participants were asked to recall the presented letters in their correct order of appearance. There were three trials per set size with different sentences (altogether 42 Items). One point was awarded for each correctly retrieved letter provided that it had also been recalled in correct position. The total score was computed by adding up the awarded points.

#### Pre-test

The pre-test measured prior PCK and PPK about multiple representations by six open-ended questions. Three questions tapped on each knowledge type (e.g., PPK aspects: “Please name three functions that can be fulfilled by using multiple representations”, “Please name typical problems that can emerge when learning with multiple representations”, “Which possibilities for support do you know when handling multiple representations?”; PCK aspects: “Please name four arbitrary representations (also graphic) for fractions”, “How many representational forms does the EIS principle comprise? Please name them”, “Do you know any other mathematic-didactic principles?”) In a blind coding, open questions were scored on the basis of a previously developed category system (for details on the category system, see Section “Category System”). Twenty per cent of the questions were rated by a second rater (high inter-rater agreement; not adjusted *ICC* = 0.97); disagreements were resolved by discussion.

#### Conditions and learning environments

The participants studied either the “integrated” or “separated” computer-based learning condition. Each condition contained information about multiple external representations from both a general pedagogical/psychological stance (i.e., PPK aspects) and a mathematics education stance (i.e., PCK aspects in the domain of fractions). Thus the crucial difference between conditions was that PPK and PCK aspects on multiple external representations were treated either interrelated or apart from one another.

The learning environments were based on several mathematical and psychological book chapters and journal articles. We based the mathematics education stance (i.e., PCK learning environment) on the German mathematics education literature that is applied later in the participants’ (and related) programs (Wittmann, 1981; Hasemann, 1986; Hefendehl-Hebeker, 1996; Zech, 1996; Padberg, 2009; Eichelmann et al., 2012). We addressed several didactic aspects worth consideration when working with multiple external representations (e.g., text, pictures, graphs, tables, etc.) in the classroom. We presented translation pitfalls between different representations of fractions (e.g., difficulties of students in locating a fraction on a number line), different aspects of fraction numbers (e.g., fraction numbers as a fraction or ratio), and, as a strategy for teaching particular topics, the EIS principle (i.e., enactive, iconic, symbolic approach to learning contents) which is included in almost all German standard books on mathematics education but is not found in the pedagogical/psychological literature used in teacher education.

The content of the general pedagogical/psychological stance (i.e., PPK learning environment) focused on different, more general aspects which should be considered when working with multiple external representations in classroom. We presented psychological functions of multiple external representations (e.g., that one representation can constrain the interpretation of another), possibilities of adequate support for students on a surface-feature level (e.g., by color-coding) or on a deep-structure level (i.e., by explicitly explaining the relations between corresponding structures), and informed on the cognitive demands of establishing a coherent mental model. The information on the pedagogical/psychological stance was based on the book chapter by Bodemer (2008) and journal articles by Ainsworth (1999, 2006), Rau et al. (2009), Schwonke et al. (2009), and Seufert and Brünken (2006).

In the separated condition, the two learning environments (i.e., on the general pedagogical/psychological stance and on the mathematics education stance) were administered in counterbalanced order (i.e., half of the participants in the separated condition worked first on the PPK environment, and half of the participants worked first on the PCK environment). Whereas the mathematics education stance used fractions as subject matter, the pedagogical/psychological stance contained illustrations with everyday-life examples (e.g., the genesis of Fata Morganas). Unlike the separated condition, the integrated condition encompassed just one learning environment. It was established by using the contents of the single learning environments and combining them in a thematically coherent sequence. To achieve closely integrated contents, examples of the pedagogical/psychological stance were related to the topic of fractions. In order to smooth transitions between stances and achieve integration of different topics (i.e., slides dealing with either pedagogical content aspects or general pedagogical/psychological aspects) some connecting phrases – not included in the separated condition – were added. These phrases did not, however, contain additional information on teaching with multiple representations (e.g., “Before the functions will be explained in detail, a brief introduction to the EIS principle will be provided”). The number of words and basic information on the two experimental conditions were kept constant. To achieve this, some text passages in the integrated condition were shortened slightly in order to balance for the additional connecting sentences mentioned previously. To ensure a standardized order of information processing in the learning program we did not allow the participants to skip back to earlier contents. In both experimental conditions, students were instructed to proceed at a pace that would enable substantial learning and the answeringof follow-up questions.

#### Post-test

The post-test consisted of ten rapid assessment items and ten open-ended questions. The rapid assessment items required the learners to indicate whether a statement was valid or invalid (i.e., rapid verification method). Rapid assessments have proven to be a valid and time-saving approach to assess knowledge (e.g., Kalyuga, 2006, 2008). All rapid verification items referred to descriptions of teaching situations that were displayed for a limited time immediately before the statements (e.g., a lesson was described in which teacher and students sort paperclips by color, then discuss different graphic representations and speak about ways to represent these distributions in tables later on; one rapid assessment item tested whether the participants recognized the central purpose from the psychological/pedagogical stance: “Function 3 is fulfilled: different representations of a concept support deep understanding.”). Rapid verification items were constructed for PCK and PPK knowledge aspects (i.e., aspects of PCK or general PPK on multiple representations).

**FIGURE 1:**
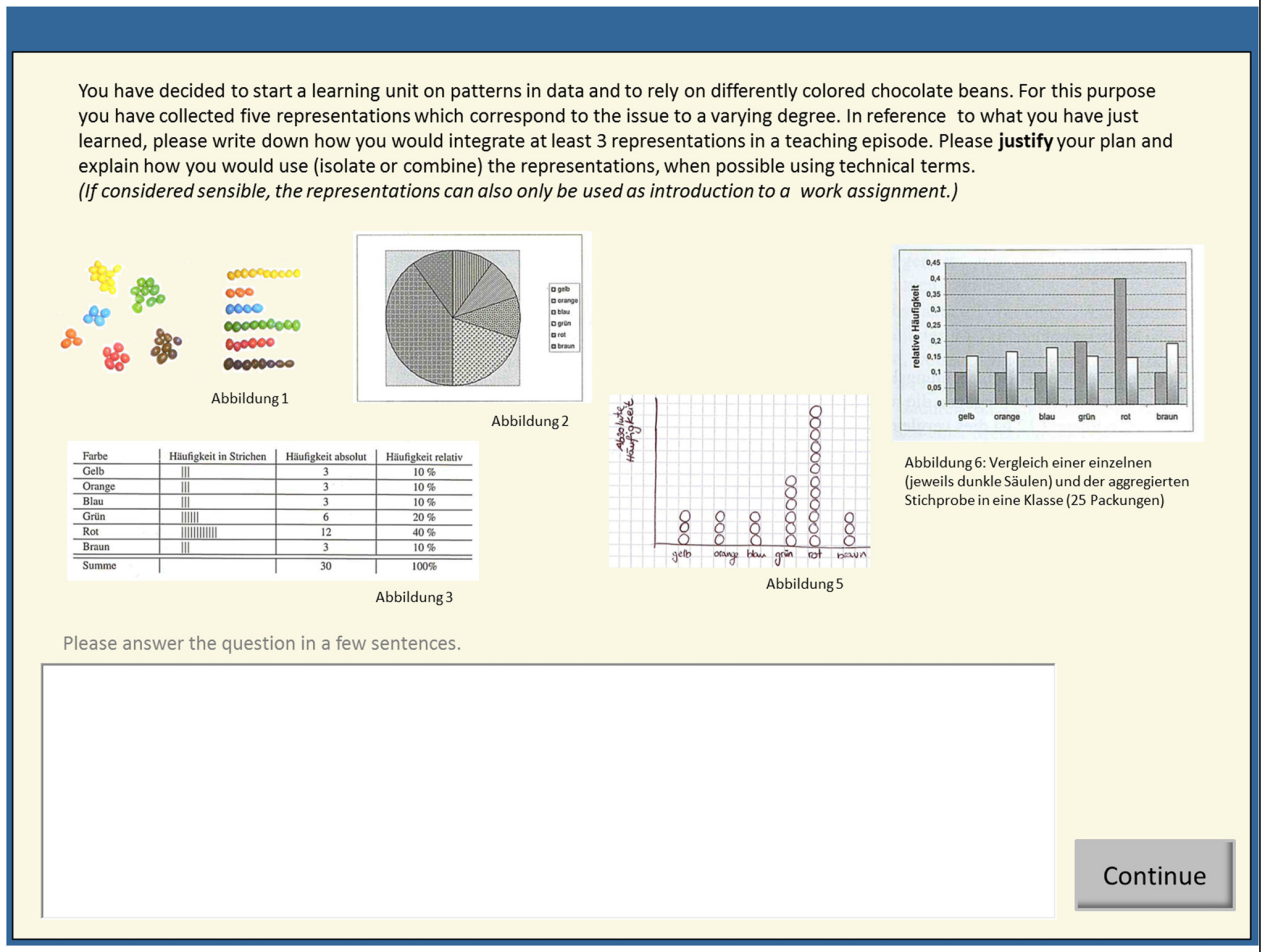
**Translated screenshot of a mathematics classroom scenario.** Student teachers completed this task individually. They were asked to combined different elements in accordance with the principles learned in the preceding learning environment(s).

Five of the open-ended questions asked for particular contents such as naming different functions of multiple external representations or listing possibilities of mathematical representations [e.g., PPK aspects: “Please name three functions that can be fulfilled by using multiple representations”; PCK aspects: “Please name four arbitrary representations (also graphic) for fractions”]. The other five open items were designed as mathematics classroom scenarios in a subdomain different from the learning subdomain (i.e., data and chance, relatively novel obligatory mathematics content in German schools; Eichler and Vogel, 2009). In order to solve the items, either aspects of PCK or general PPK, or both knowledge types could be sensibly applied (e.g., a scenario was described in which the participants assumed the teacher’s role and started a learning unit addressing patterns in data. The task’s starting situation was that the teacher had already decided to use colored chocolate beans for illustration purposes and had collected five tables and illustrations (of which at least three had to be used when sketching a teaching episode). Students were asked to justify their answer; for further details please see **Figure 1**). As with the pre-test, the post-test score was assessed using a blinded coding system, applying a category scheme distinguishing different performance levels (for details on the category system, see Section “Category System”). We determined two scores - one for PCK aspects (*Cronbachs alpha* = 0.63) and one for general PPK aspects (*Cronbachs alpha* = 0.63). Twenty per cent of the questions were rated by a second rater (high inter-rater agreement; not adjusted *ICC* = 0.91). Disagreements were resolved by discussion. The measure of a combined use of knowledge types was obtained according to the scores for PCK and general PPK by awarding a point for each mathematics classroom scenario solved by using both knowledge types (i.e., PCK and PPK aspects on multiple external representations) instead of using only one.

### PROCEDURE

In both experimental conditions, the participants first received brief information on how to work with the computer program used in the experimental session. Then, they were asked to proceed to the computer-based working memory task. After completing this task, participants worked on the pre-test on prior PCK and PPK. They then received information on the topic of the learning phase and on the post-test questions. The participants were furthermore informed that learning would be self-paced and that they could monitor their progress by looking at a progress bar located in the upper left corner in each slide. Following this information, they worked on the learning environments in the two conditions at their own pace. Finally, the participants completed the post-test.

### CATEGORY SYSTEM

The open-ended pre-test and post-test questions were scored on the basis of a category system assessing aspects of PCK and general PPK. We relied on expert answers containing aspects of both knowledge types of the learning environments (i.e., a psychological/pedagogical stance and mathematics education stance). The answers were broken down into single statements addressing different aspects of knowledge. Participants were allocated a point for each correct statement. We coded statements that involved a general pedagogical/psychological stance (e.g., functions of multiple external representations or possibilities of adequate support) as PPK aspects. In contrast, statements indicating the use of a mathematics education stance (e.g., typical pitfalls or the EIS-principle as a strategy for teaching the particular content) were coded as PCK aspects (for exemplary excerpts of students answers see **Table 2**). If the same aspect was mentioned several times in response to one item, no additional points were allocated. As the complexity levels differed between items and a various number of aspects could be reasonably applied, the maximum scores varied between items, with six the maximum. To equalize the weight of the single items, all item scores were z-standardized before aggregation. In the final scoring process, scores of open-ended questions and rapid assessment were summed up to create the scales for PCK and general PPK.

**Table 2:** Exemplary excerpts of student answers coded as PPK and PCK aspects.

PPK	PCK
“To achieve a constraint of interpretation [= a function of multiple external representations], element 4 [pie chart] can be depicted with chewing gum obtained in element 2 [gumdrops of different colors]”	“The high school students would be given the task to sort, arrange and count a specific number of chocolate drops [enactive] in order to draw a bar chart [iconic] and then draw a table of absolute and relative frequencies [symbolic], in a further step”

## RESULTS

**Table 3** provides an overview of the mean scores and standard deviations of central variables for the experimental conditions. For all statistical comparisons we used an alpha level of 0.05. As an effect size measure, Cohen’s *d* was used. According to Cohen (1988) values of 0.2 were labeled as small effects, values between 0.5 and 0.8 as medium effects, and values >0.8 as large effects. Due to missing data, sample size varies between 58 and 59 participants in the following analyses.

**Table 3:** Means (and SD) of important variables in the two experimental groups.

		Integrated condition	Separated condition	Effect size Cohen’s *d*
Working memory		0.08 (0.98)	–0.08 (1.03)	0.16
Pre-test	Overall score	0.19 (0.93)	–0.21 (1.05)	0.41
Post-test	Pedagogical/psychological knowledge	0.38 (0.90)	–0.40 (0.95)	0.86**
	Pedagogical content knowledge	0.01 (0.95)	–0.02 (1.07)	0.17
	Combined use of knowledge	0.33 (1.02)	–0.36 (0.86)	0.74**
Learning time in min		23.85 (4.77)	23.76 (7.17)	0.01
Post-test time in min		28.21 (9.55)	28.36 (9.07)	0.02

**p < 0.01.

First, we tested potential group difference in prior knowledge, working memory capacity and demographic variables such as age, number of attended pedagogic courses, number of semesters, and mother tongue. We also checked learning time differences. We did not find significant group differences (all *p*s > 0.10).

Hypothesis 1 predicted that participants can apply general PPK better if they learn general PPK and PCK in an integrated way. We observed a significant effect of condition on aspects of general PPK, *t*(56) = 3.21, *p*(one-tailed) = 0.001, *d* = 0.86. Despite the randomization, we observed that the integrated group had slightly (non-significant) higher scores in both, PPK and PCK aspects. Hence, we planned to confirm the effect on PPK aspects via ANCOVA controlling for prior knowledge. However, prior knowledge did not significantly predict PPK aspects (*r* = 0.19, *p*(one-tailed) = 0.075), hence it made no sense to compute an ANCOVA model. Overall, the group differences in PPK aspects were obviously not due to *a priori* differences. An additional analysis to assess a potential impact of integration on PCK aspects revealed no significant effect between the integrated and separated conditions, *t*(56) = 0.12, *p*(two-tailed) = 0.909, *d* = 0.17. To account for the integrated group’s slight yet non-significant advantage on prior knowledge, we confirmed the finding on PCK aspects by an ANCOVA controlling for prior knowledge, *F*(1,55) = 0.35, *p* = 0.557, *d* = 0.17. As we only expected an effect on aspects of general PPK, Hypothesis 1 was confirmed.

Hypothesis 2 predicted that the integrated group should be superior in applying both perspectives simultaneously to specific classroom situations. Actually, the integrated group significantly outperformed the separated group, *t*(56) = 2.78, *p*(one-tailed) = 0.004, *d* = 0.74. To account for slight prior knowledge differences, we confirmed the effect via ANCOVA controlling for prior knowledge, *F*(1,55) = 5.28, *p* = 0.025, *d* = 0.74. Hypothesis 2 was thus confirmed.

Finally, we tested Hypotheses 3 and 4 predicting that the positive effects of integrated presentation are moderated by prior knowledge (Hypothesis 3) and working memory capacity (Hypothesis 4). We found no moderating effects of prior knowledge (interaction term of group and prior knowledge: *F*(1,54) = 0.08, *p* = 0.775) or working memory capacity (interaction term of group and working memory capacity: *F*(1,54) = 0.50, *p* = 0.484) for knowledge application. Hypotheses 3 and 4 were thus rejected.

## DISCUSSION

We investigated the impact of integrating different knowledge types that are usually taught separately in teacher education (Ball, 2000). We used two different learning conditions, each containing information about multiple external representations from a general pedagogical/psychological stance (e.g., Ainsworth, 1999) as well as from a mathematics education stance (e.g., Padberg, 2009). Participants were mathematics student teachers with only little prior knowledge on PPK and PCK related to learning from multiple representations. We formulated several hypotheses addressing different aspects of knowledge application and moderating factors. Our findings can be summarized as follows: First, we found that integrated presentation is an effective means of increasing the applicability of PPK aspects. As predicted, participants who received the integrated perspective of pedagogical/psychological principles and of corresponding mathematics education (on the use and handling of multiple external representations) clearly applied more aspects of general PPK. Furthermore, the integrated presentation did not impair the application of the PCK aspects that had been acquired. As suggested by Renkl’s (2014) theory of example-based learning and Bandura’s (1986) sub-theory of abstract modeling (1986) participants learning from an integrated presentation were able to apply general PPK to a greater extent (see **Table 3**). If such a shift in application of teaching relevant concepts (Voss et al., 2011) could be achieved in university courses, it should yield major benefits for the quality of education: Inert knowledge (Whitehead, 1929; Renkl et al., 1996) could be prevented (i.e., a non-use of PPK when teaching particular content areas) and negative consequences for teaching processes avoided. In short, teachers would be enabled to draw on larger parts of their knowledge for lesson planning and implementation.

Second, in line with our preceding result, an integrated presentation increased simultaneous application of aspects of both knowledge types when solving a particular problem in classroom teaching. Accordingly, participants who were provided with the integrated learning condition more often applied both perspectives (i.e., aspects of PPK and PCK) simultaneously than participants who were provided with the separated learning condition. As suggested by Anderson’s (1983) assumption of spreading activation fostering an integrated perspective of knowledge types proved beneficial for simultaneously applying different perspectives to solve a specific task. Thus, it can be assumed that an integrated presentation of different teacher education disciplines (i.e., subject and pedagogy) should promote an increased retrieval of PPK when PCK contents are activated in classroom settings.

Third, and in contrast to our predictions, low prior knowledge did *not* reduce the effect of the integrated presentation, which is “good news”. Fourth, and analogous to the findings on prior knowledge, neither did working memory capacity moderate the effect of knowledge integration. In summary, learning conditions did not possess differential properties depending on prior knowledge or working memory capacity. Against reasonable worries concerning overloading of learners capacity (e.g., Sweller et al., 2011; Ayres, 2013) the integrated, complex, and demanding presentation obviously did not overwhelm participants with low prior knowledge or low working memory capacity.

What further implications can be drawn from these findings? With regard to teacher education, our findings can be considered a hint that segregating pedagogical/psychological courses from content-related courses (i.e., courses teaching PCK) is suboptimal. This implication is also supported by related research. Similar to our question concerning integration of pedagogical knowledge, a framework called technological PCK (short: TPACK) evolved over the past decade. The TPACK framework addresses the question of effective technology integration (Mishra and Koehler, 2006; Koehler et al., 2013; Tondeur et al., 2013). Here as well, the “default approach” in higher education institutions is to relegate technology to separated courses (Kay, 2006; Koehler et al., 2014). These courses are typically taught by an instructional technologist with either restricted expertise in all content areas or the goal to broadly cover technology overarching all content areas. Comparable to PPK, student teachers are responsible for the integration of knowledge types (Koehler et al., 2014). Nevertheless, regarding technology integration two additional approaches exist. The second approach is focused on an integration of PCK and TPACK. Here, PCK already developed through methods courses and experience gets later enriched by technology (e.g., Harris and Hofer, 2009; Niess et al., 2010). However, the success of this approach is limited due to the unwillingness of in-service teachers, who “already know how to teach”, to try new technology-supported strategies (Niess et al., 2010). The third approach is focused on a simultaneous acquisition of knowledge types. In contrast to the other approaches, this pathway is actually working with a systematic integration of technology in content specific methods courses (Koehler et al., 2014). Thus, a program following this approach might, for example, not have an overarching technology course but rather demand that content-specific methods courses include how to use technology in a particular subject area. Hence, a direct connection to the specific teaching subject is made. Research concerning the effectiveness of this approach shows that by this means a significant increase of technology-related knowledge and richer conceptions emphasizing connections among knowledge types are achieved (Koehler and Mishra, 2005; Koehler et al., 2007).

In line with the TPACK framework our findings indicate a potential benefit from integrating courses. However, our findings must be interpreted with caution. We conducted a short-term experimental study in which we focused on some instructionally relevant subdimensions of general PPK and PCK. Conclusions regarding long-term processes in teacher education can hence only be tentative.

The need for explicitly fostering applicable knowledge in education is highlighted by several further traditions (e.g., inert knowledge research, e.g., Renkl et al., 1996; transfer research, e.g., Goldstone and Day, 2012) and representatives of different institutions (e.g., teacher educators, researchers, and politicians, e.g., Ball, 2000; Grossman et al., 2009; Seidel et al., 2013). In this regard, research traditions commonly share the understanding that bringing different parts of knowledge together (e.g., pedagogical/psychological principles of handling multiple external representations and information on how these representations are used in a particular content domain) is beneficial for transferable knowledge (e.g., Renkl, 1997; Ross and Kilbane, 1997; Renkl and Atkinson, 2007; Colhoun et al., 2008; Goldstone and Wilensky, 2008). Our results are in line with this understanding and yield initial empirical evidence for the widespread assumption of integration benefits in the particular case of teacher education. In a nutshell, our findings provide hints that the integration of knowledge types is a promising approach in teacher education and that such a change in presenting university knowledge might actually pave the way to more effective application of general pedagogical/psychological principles.

### LIMITATIONS AND IMPLICATIONS FOR FURTHER RESEARCH

As already mentioned, the present findings are based on a short-term experimental study in which we focused on specific subdimensions of general PPK and PCK when handling multiple external representations (a central issue in mathematics). Due to limited time and limited generality, our findings are only tentative implications for long-term processes in teacher education. However, this limitation of our approach can also be regarded as a potential strength. Due to standardized procedure and instruction as well as randomization process, confounding variables can be controlled and effects can be attributed to the experimental variation. Still, a remaining restriction is that due to the specificity of the content no previously validated instruments could be used. We conducted our study as an initial examination of integration effects that can provide a sound basis for a long-term study. Moreover, our study contributes to deeper understanding of the acquisition and application of PPK. Nonetheless, it would certainly be worthwhile to further explore the relationship between integrating knowledge types and the applicability of pedagogical/psychological principles on a longer timescale using traditional survey methods with tested construct validity.

A problem not addressed in our study is how to restructure university curricula so that student teachers can be taught different knowledge types in integrated courses. For this objective, teacher educators of different disciplines (i.e., of subject and educational courses) would have to get involved in a mutual discourse to gage overlapping information of both disciplines and match content which would benefit from a connection with pedagogical knowledge and vice versa. To illustrate such a benefit of integration one could imagine a university course where not only fractions are discussed but at the same time the obstacles they pose for students are addressed as well as corresponding means of support. Unfortunately, the separation/segregation of courses has a long tradition (Ball, 2000). We are well aware that it will not be abandoned easily. However, since the beneficial effects are attributed to the integrated encoding of knowledge types, an investigation of other possibilities to foster such integrated encoding would seem useful. We see a promising means of integration in special homework assignments or tutorials. Of course, a discourse of teacher education disciplines about useful links would be substantial as well; however, courses could remain separated and substantial change of curricula would be unnecessary. In this respect, we plan to examine benefits of cue cards which could be used to trigger mental integration processes that often fail to occur spontaneously when material is presented separately (Gentner et al., 2009). By this means, courses could continue to remain formally separated but get mentally integrated. However, this assumption must undergo further research. In this respect, our study can be regarded as a promising first step towards future investigation.

## Conflict of Interest Statement

The authors declare that the research was conducted in the absence of any commercial or financial relationships that could be construed as a potential conflict of interest.
